# Artificial Spores: Immunoprotective Nanocoating of Red Blood Cells with Supramolecular Ferric Ion-Tannic Acid Complex

**DOI:** 10.3390/polym9040140

**Published:** 2017-04-13

**Authors:** Taegyun Park, Ji Yup Kim, Hyeoncheol Cho, Hee Chul Moon, Beom Jin Kim, Ji Hun Park, Daewha Hong, Joonhong Park, Insung S. Choi

**Affiliations:** 1 Center for Cell-Encapsulation Research, Department of Chemistry, Korea Advanced Institute of Science and Technology (KAIST), Daejeon 34141, Korea; xorbs7467@kaist.ac.kr (T.P.); y123812@kaist.ac.kr (J.Y.K.); harry0305@kaist.ac.kr (H.C.); arbalest@kaist.ac.kr (H.C.M.); kimbj20@kaist.ac.kr (B.J.K.); pjh1987@kaist.ac.kr (J.H.P.); 2 Department of Chemistry and Chemistry Institute of Functional Materials, Pusan National University, Busan 46241, Korea; dwhong17@pusan.ac.kr; 3 Department of Laboratory Medicine, College of Medicine, The Catholic University of Korea, St. Mary’s Hospital, Daejeon 34943, Korea; miziro@catholic.ac.kr

**Keywords:** artificial spores, cell-surface engineering, immunoprotection, nanocoating, red blood cells, supramolecular complex

## Abstract

The blood-type-mismatch problem, in addition to shortage of blood donation, in blood transfusion has prompted the researchers to develop universal blood that does not require blood typing. In this work, the “cell-in-shell” (i.e., artificial spore) approach is utilized to shield the immune-provoking epitopes on the surface of red blood cells (RBCs). Individual RBCs are successfully coated with supramolecular metal-organic coordination complex of ferric ion (Fe^III^) and tannic acid (TA). The use of isotonic saline (0.85% NaCl) is found to be critical in the formation of stable, reasonably thick (20 nm) shells on RBCs without any aggregation and hemolysis. The formed “RBC-in-shell” structures maintain their original shapes, and effectively attenuate the antibody-mediated agglutination. Moreover, the oxygen-carrying capability of RBCs is not deteriorated after shell formation. This work suggests a simple but fast method for generating immune-camouflaged RBCs, which would contribute to the development of universal blood.

## 1. Introduction

**Figure 1:**
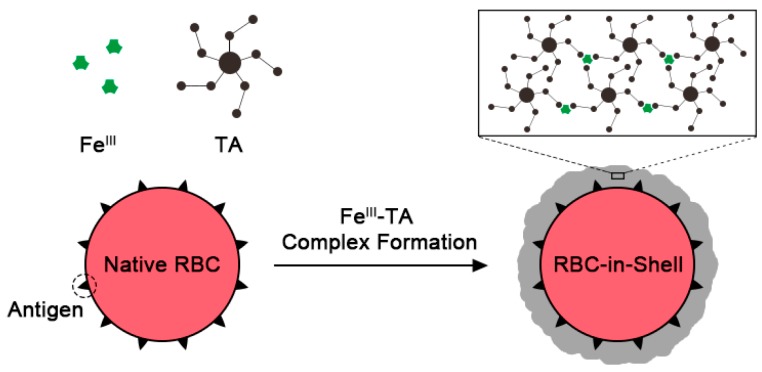
Schematic representation for supramolecular shell formation of ferric ion (Fe^III^) and tannic acid (TA) on individual red blood cells (RBCs).

The “cell-in-shell” structures (a.k.a., artificial spores [1,2,3] or micrometric Iron Men [4]) are the emerging cell hybrid entities in biomedical and nanomedicinal fields, where individual live cells are encapsulated within nanometric (<100 nm) shells. Microbial and mammalian cells have been coated with silica, silica-titania, polymers, and metal-organic frameworks, and the cells inside are protected from the harmful, and often lethal, attack of enzymes, nanoparticles, heat, or UV light [5,6,7,8,9,10,11,12]. Live cells also have been interfaced or three-dimensionally confined with liposomes [13], carbon nanotubes [14], and graphene [15,16]. The shell formation and degradation are further controlled chemically, which allows for temporal cytoprotection of therapeutically functional cells during in vitro manipulation and storage, inspired by sporulation and germination processes found in nature [12,17,18,19,20]. Among the functional cells employed so far in the field of artificial spores, red blood cells (RBCs) would be one of the simplest but highly important cells in cell therapy and related fields [21]. RBCs are the anucleate, non-dividing cells, the main role of which is oxygen delivery in the body. Approximately 85 million units of RBCs are transfused annually worldwide [22], but blood supply, mostly supported by donation, falls short of the demand. Blood-type mismatch poses a worse problem in transfusion medicine, causing antibody-mediated immune responses, such as cross-type agglutination and hemolysis, which lead to life-threatening situations [23]. To tackle these problems in blood transfusion, immune-camouflaged “RBC-in-shell” structures have been proposed as a universal blood, where the immune-provoking epitopes on RBC surfaces are shielded by the encasing shells. The approach of artificial spores would be much simpler and more cost-effective than other strategies, such as enzymatic cleavage of the antigens [24] and biological production of O (Rh^+^) RBCs from hematopoietic stem cells [25,26]. Moreover, the approach of enzymatic cleavage can be applied only to A and B antigens, not to the protein-based D (Rh) antigen, because glycosidases are mainly used for the cleavage reactions [27]; the in vitro RBC production is difficult to scale up [28]. RBCs have been coated with polyelectrolyte multilayers (PEMs) by multi-step layer-by-layer (LbL) assembly for the attenuation of antigen-antibody recognition [29]. One-step coating of polydopamine [30] or plant-derived pyrogallol [31] also has recently been applied for immunoprotection of RBCs. However, all the reported methods require hours of coating steps, which precludes the clinical applications. In this paper, we report a simple but rapid method for fabricating immune-camouflaged RBCs, based on the supramolecular metal-organic coordination complex of ferric ion (Fe^III^) and tannic acid (TA) (Figure 1).

## 2. Results and Discussion

TA is a natural polyphenol, which has several galloyl groups attached to a glucose molecule. Because the galloyl groups of TA form multivalent coordination bonds with Fe^III^, TA molecules are cross-linked by Fe^III^ to form a supramolecular metal-organic Fe^III^-TA complex (Fe^III^-TA-MOC). The universal surface-binding affinity of TA (and its assemblies) allows for the Fe^III^-TA nanofilm formation on various substrates including planar or particulate substrates having different surface properties [32,33]. We and others have previously demonstrated that the material-independent Fe^III^-TA nanocoating method could be utilized to coat individual living cells, such as yeast, *Escherichia coli*, HeLa, NIH 3T3, PC-12, and Jurkat cells, without significant loss of cell viability [17,18,20]. However, this method cannot be directly applied to RBCs, because RBCs are extremely sensitive to the tonicity of the reaction solutions and chemicals. In this work, we optimized the coating conditions of the supramolecular Fe^III^-TA-MOC for RBC coating and successfully generated “RBC-in-shell” structures.

We used the isotonic 0.85% NaCl solution during the entire coating processes including washing. RBCs were found to be swollen and hemolyzed rapidly in deionized (DI) water (data not shown). We also optimized the concentration of TA and found that 0.05 mg mL^−1^ was optimal for RBCs; above that concentration were clumped RBCs (Figure S1). The mass ratio of TA to FeCl_3_∙6H_2_O was fixed to 4:1 for uniform coating according to the previous studies [17,32]. Briefly, to the RBC suspension in the 0.85% NaCl solution were added the TA stock solution and the FeCl_3_∙6H_2_O stock solution (final concentration: [TA] = 0.05 mg mL^−1^, [Fe^III^] = 0.0125 mg mL^−1^) sequentially, with 10-second mixing after each addition. 3-(*N*-morpholino)propanesulfonic acid (MOPS)-buffered saline (pH 7.4) was then added for pH stabilization, which made Fe^III^-TA-MOC stable. The whole coating processes were conducted in one pot for less than one minute, and repeated four times to generate the Fe^III^-TA-coated RBC (RBC@[Fe^III^-TA]). We found that the addition sequence of TA and Fe^III^ was critical in the stable formation of Fe^III^-TA coats. When the Fe^III^ stock solution (>0.001 mg mL^−1^) was added to the RBC suspension, RBCs aggregated uncontrollably (Figure S2), presumably because of multivalent interactions of metallic cations with RBCs [34]. However, when the TA stock solution was added first to the RBC suspension, individual RBCs were successfully coated with Fe^III^-TA-MOC without agglutination. We think that the pre-deposited TA molecules rapidly formed Fe^III^-TA-MOC, when Fe^III^ was added; the Fe^III^-TA-MOC formation on RBC surfaces would decrease effective Fe^III^ concentration in solution, and, additionally, the Fe^III^-TA-MOC further bound with free TA in solution.

**Figure 2:**
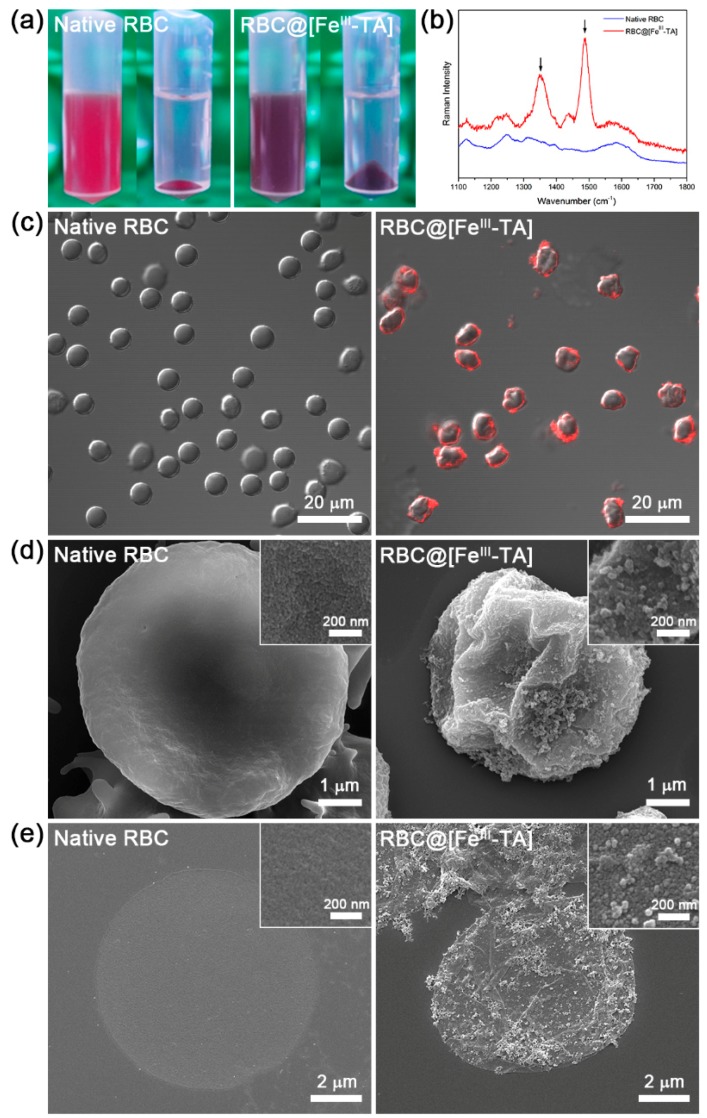
Characterizations of native RBC and RBC@[Fe^III^-TA]. (**a**) Photographs of RBC suspension and pellet before and after shell formation. (**b**) Raman spectra of (blue) native RBC and (red) RBC@[Fe^III^-TA]. Black arrows indicate the strong bands attributed to the ring structures of TA. (**c**) CLSM images of native RBC and RBC@[Fe^III^-TA] after incubation with BSA-Alexa Fluor^®^ 647. (**d**) SEM micrographs of native RBC and RBC@[Fe^III^-TA]. (**e**) SEM micrographs of the membrane of native RBC and RBC@[Fe^III^-TA] after hypotonic lysis.

The isotonic saline solution of RBC@[Fe^III^-TA] was changed from red to dark purple in color, indicating that the Fe^III^-TA coat was successfully formed (Figure 2a). We also visualized the Fe^III^-TA coat by adsorbing Alexa Fluor^®^ 647-conjugated bovine serum albumin (BSA-Alexa Fluor^®^ 647) onto RBC@[Fe^III^-TA] (Figure 2c). The confocal laser-scanning microscopy (CLSM) images clearly showed red-fluorescent rings only for RBC@[Fe^III^-TA]. When we treated RBCs only with TA, we did not observe any fluorescence (Figure S3). We characterized RBC@[Fe^III^-TA] by Raman spectroscopy, scanning electron microscopy (SEM), transmission electron microscopy (TEM), and atomic force microscopy (AFM). The Raman spectrum of RBC@[Fe^III^-TA] showed intense bands at 1349 and 1487 cm^−1^, corresponding to C-C ring vibration and C-H bending of TA (Figure 2b). In the SEM micrographs, the surface became rougher and more grainy after coating than the surface of native RBCs (Figure 2d). Although RBCs were crumpled after Fe^III^-TA coating, any defects or fractures were not observed. To further characterize the cell membranes in the SEM images, we immersed RBCs (native or coated) in DI water for hypotonic lysis and air-dried them on a flat silicon wafer. Compared with the smooth surface of lysed native RBCs, the surfaces of lysed RBC@[Fe^III^-TA] cells were grainy, indicating that the Fe^III^-TA coat was not affected greatly by hypotonic lysis (Figure 2e). On a closer view, many wrinkles were clearly observed only on the membrane of lysed RBC@[Fe^III^-TA] cells. These results suggested that the Fe^III^-TA coat reinforced the integrity of RBC membranes; it has been well reported that hollow capsules are folded when they collapse, unless their capsule shells have defects [32,35].

**Figure 3:**
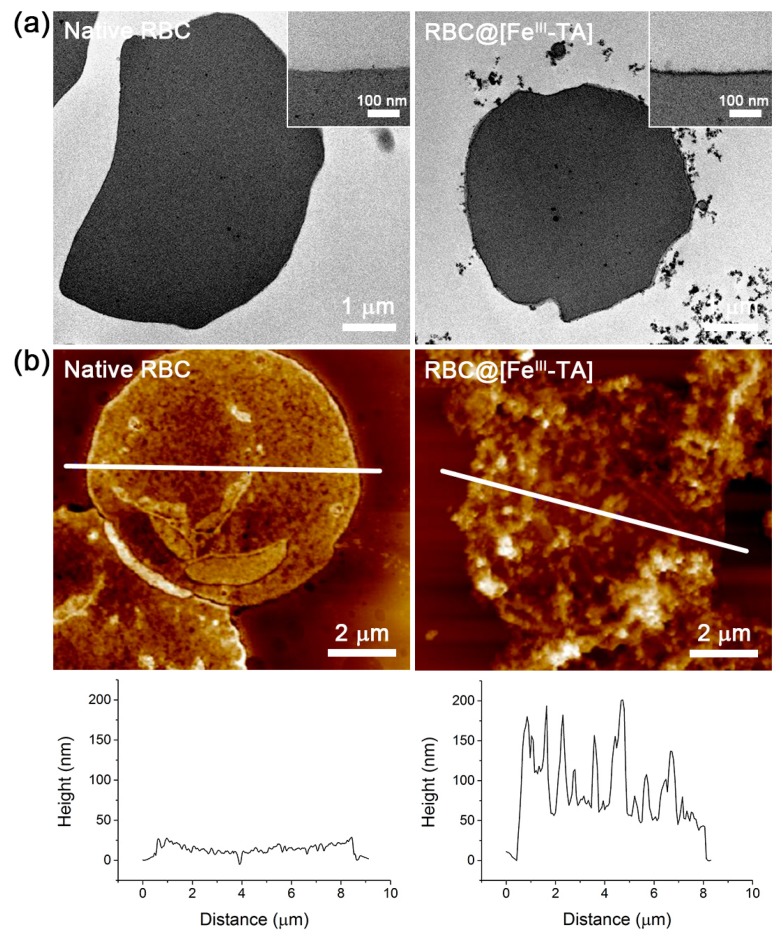
(**a**) TEM micrographs of native RBC and RBC@[Fe^III^-TA]. (**b**) (**top**) AFM micrographs and (**bottom**) line-profile graphs of native RBC and RBC@[Fe^III^-TA]. White lines in AFM micrographs indicate the path of line-profile analysis.

The uniform Fe^III^-TA coat was clearly observed in the TEM images of microtomed RBC@[Fe^III^-TA], and the average thickness was measured to be around 20 nm (Figure 3a). The thickness of the Fe^III^-TA coat was also estimated by the AFM line-profile analysis. The minimum heights of the collapsed membranes were measured to be about 10 and 50 nm for native RBC and RBC@[Fe^III^-TA], respectively (Figure 3b). Therefore, the film thickness was calculated to be about 20 nm, which was in agreement with the results from the TEM analysis. It is to note that the relatively thick films (5 nm per coating) were formed with the use of low concentrations of TA and Fe^III^ (final concentration: [TA] = 0.05 mg mL^−1^; [Fe^III^] = 0.0125 mg mL^−1^). For comparison, the previous reports with polystyrene particles showed that the 10-nm-thick film was formed with 0.4 mg mL^−1^ of TA and 0.1 mg mL^−1^ of Fe^III^ [32]. The thick film formation in this work was attributed to the use of isotonic saline instead of DI water, because we found that NaCl increased the film thickness with gold substrates as a model (Figure S4). In the model studies, the gold substrates were coated with Fe^III^-TA-MOC in DI water or isotonic saline ([TA] = 0.05 mg mL^−1^; [Fe^III^] = 0.0125 mg mL^−1^), and the film thickness was measured by ellipsometry. The ellipsometric measurements indicated that the films formed in isotonic saline were 3.4 times thicker than those in DI water. The effects of ions on film thickness in the Fe^III^-TA coating is currently being investigated in detail.

**Figure 4:**
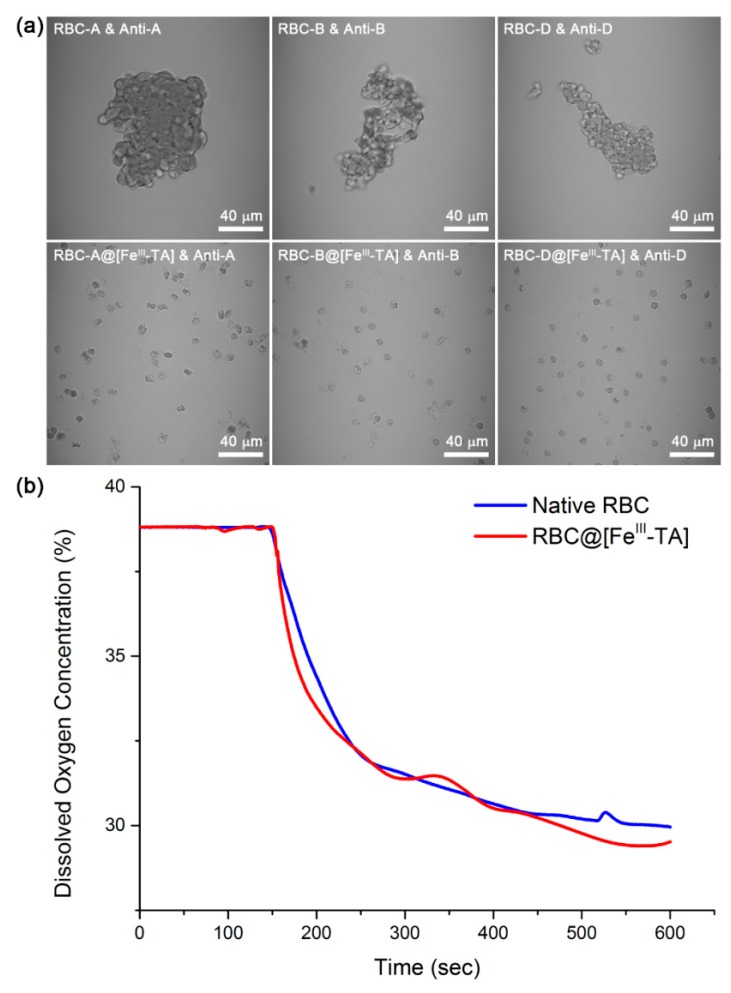
(**a**) Antibody-mediated agglutination assay. Native RBCs or RBC@[Fe^III^-TA] cells were mixed with their anti-type sera, and the optical images were taken after one hour. (**b**) Oxygen consumption graphs. The dissolved oxygen concentration (%) in PBS (pH 7.4) was plotted as a function of time. The initial oxygen concentration dissolved in the O_2_-purged PBS was about 39%, and the oxygen concentration was recorded by the oxygen probe connected with LabQuest^®^.

To assess the immunoprotective effect of Fe^III^-TA coats, we performed the antibody-mediated agglutination assay. Because ABO/D blood typing should be conducted before blood transfusion, RBCs of type A, B, and D (Rh) were used for the assay. Each type of RBCs is determined by the presence of specific surface antigens, which causes agglutination with the anti-type antibodies. When treated with their anti-type sera (anti-A, anti-B, or anti-D (Rh)), RBC@[Fe^III^-TA] remained unaffected, while native RBCs harshly agglutinated (Figure 4a). The results indicated that the Fe^III^-TA coat was uniformly formed on all types of RBCs and successfully prevented the access of antibodies to the RBC surface-antigens, acting as an immunoprotective barrier. Oxygen transport is a vital function of RBCs in the body, and, therefore, many immunoprotective strategies have tried to maintain the oxygen-carrying capacity of native RBCs after modification for the development of universal RBCs. To investigate the oxygen-carrying property of “RBC-in-shell” structures, native or coated RBCs were added to an O_2_-purged (initial oxygen concentration: ~39%) phosphate-buffered saline (PBS, 10 mM, pH 7.4), and the dissolved oxygen concentration was monitored over time with an oxygen probe (Figure 4b). Two time-lapse graphs for native RBC and RBC@[Fe^III^-TA] had similar shapes to each other, and there was no significant difference in the amount of oxygen consumption after saturation (8.9% for native RBC and 9.4% for RBC@[Fe^III^-TA]), indicating that the Fe^III^-TA coat did not inhibit oxygen diffusion and hemoglobin function.

## 3. Experimental Section

### 3.1. Materials

Tannic acid (TA, Sigma), iron(III) chloride hexahydrate (FeCl_3_∙6H_2_O, Sigma, St. Louis, MO, USA), 3-(*N*-morpholino)propanesulfonic acid (MOPS, Sigma, St. Louis, MO, USA), sodium chloride (NaCl, Daejung, Siheung, Korea), Alexa Fluor^®^ 647-conjugated albumin from bovine serum (BSA-Alexa Fluor^®^ 647, Life Technologies, Carlsbad, CA, USA), phosphate-buffered saline (PBS, pH 7.4, Sigma, St. Louis, MO, USA) were used as received. Anti-A, anti-B, and anti-D (Rh) antisera were purchased from Asan Pharmaceutical (Seoul, Korea). Ultrapure water (18.3 MΩ·cm) from the Human Ultrapure System (Human Corp., Seoul, Korea) was used.

### 3.2. Red Blood Cell (RBC) Samples

Blood samples were obtained after completion of clinical testing. All studied samples were collected from human subjects who provided the written informed consent, and the study protocol was approved by the Institutional Review Board of the Catholic University of Korea. The collected blood was centrifuged at 600 g for 5 min at room temperature. The plasma and the buffy coat at the top were removed by a pipette, and the remaining red blood cells were washed twice with 0.85% (*w*/*v*) NaCl.

### 3.3. RBC Coating with Fe^III^-TA Complex

Washed RBCs were re-suspended in 490 μL of 0.85% NaCl to be 1% hematocrit. The 5 μL of TA stock solution (5 mg mL^−1^ in 0.85% NaCl) and the 5 μL of FeCl_3_∙6H_2_O stock solution (1.25 mg mL^−1^ in 0.85% NaCl) were added sequentially to the RBC suspension with 10 s mixing between the additions (final concentration: [TA] = 0.05 mg mL^−1^; [Fe^III^] = 0.0125 mg mL^−1^). The resulting suspension was mixed for 10 s, and, after addition of 500 μL of MOPS-buffered saline (20 mM MOPS, 0.85% NaCl, pH 7.4) for pH stabilization, washed three times with 0.85% NaCl to remove any remaining TA and FeCl_3_, leading to the formation of single-coated RBCs. The coating processes, from addition of TA to washing, were repeated four times to generate quadruple-coated RBC@[Fe^III^-TA]. To visualize the Fe^III^-TA coat, native RBCs or RBC@[Fe^III^-TA] cells were suspended in 200 μL of the BSA-Alexa Fluor^®^ 647 solution (0.4 mg mL^−1^ in 0.85% NaCl), and the resulting suspension was incubated for 15 min at room temperature. After washing with 0.85% NaCl, the samples were characterized by confocal laser-scanning microscopy (LSM 700, Carl Zeiss, Oberkochen, Germany). To measure the film thickness, native RBCs or RBC@[Fe^III^-TA] cells were lysed by immersing them in DI water. The suspension was centrifuged at 3000 g for 30 s, and the supernatant was removed. After washing twice with DI water, the membranes of lysed native RBC or RBC@[Fe^III^-TA] were re-suspended in DI water and dropped onto a piece of silicon wafer (5 mm × 5 mm). After drying the samples in the air, they were analyzed by atomic force microscopy (Innova, Bruker, Billerica, MA, USA). Typical scans were conducted in tapping mode with OTESPA silicon cantilevers (Bruker, Billerica, MA, USA), and the coat thickness was analyzed by using line-profile data.

### 3.4. Effect of NaCl on Film Thickness: Gold Substrates

Gold-coated silicon wafers were cut into approximately 1 cm × 1 cm slides, and cleaned by sonication in acetone and ethanol, prior to use. The cleaned slides were dried under Ar gas and soaked in DI water or isotonic saline solution in a 12-well plate. The stock solutions of TA and FeCl_3_·6H_2_O were sequentially added to yield final concentrations of 0.05 and 0.0125 mg mL^−1^, respectively. The pH of the solution was then raised to ca. 8 by adding 1 M NaOH solution. After 10 s of incubation, the coated slides were washed with DI water and dried under Ar gas. The coating process was repeated four times, and the film thickness was measured with an L116s ellipsometer (Gaertner Scientific Corporation, Skokie, IL, USA).

### 3.5. Characterizations

Raman spectra were obtained with a LabRAM ARAMIS spectrometer (HORIBA Jobin Yvon, Edison, NJ, USA). The 633-nm line of an air-cooled He/Ne laser was used as an excitation source. Field-emission scanning electron microscopy (FE-SEM) imaging was performed with a Philips XN30FEG microscope (FEI-Philips Co., Hillsboro, OR, USA) with an accelerating voltage of 10 kV, after sputter-coating with platinum. Transmission electron microscopy (TEM) imaging was performed with an LEO 912AB microscope (Carl Zeiss, Oberkochen, Germany). Specimens were fixed with glutaraldehyde and paraformaldehyde, and then dehydrated in ethanol. The fixed samples were embedded in Epon 812 resin. Thin sections were cut by using ULTRACUT UCT ultramicrotome (Leica, Wetzlar, Germany) and stained with uranyl acetate and lead citrate.

### 3.6. Antibody-Mediated Agglutination and Oxygen Consumption

The immunoprotective capability of Fe^III^-TA coating was evaluated by investigating the attenuation of antibody-mediated agglutination of RBCs. The native RBC or RBC@[Fe^III^-TA] (1% hematocrit in PBS) were mixed with their anti-type sera (10:1, *v*/*v*), and the images were taken by optical microscopy (LSM 700, Carl Zeiss, Oberkochen, Germany) after one hour. The oxygen-carrying capacity of native RBC and RBC@[Fe^III^-TA] was examined by measuring the amount of oxygen uptake. The initial oxygen concentration dissolved in the O_2_-purged PBS was about 39%, and the oxygen concentration was recorded by an oxygen probe connected with LabQuest^®^ (Vernier, Beaverton, OR, USA). The probe was calibrated with the sodium sulfite calibration solution (Vernier, Beaverton, OR, USA), prior to measurement, and immersed in 2 mL of O_2_-purged PBS. Once the signal was stabilized, a suspension of native RBC or RBC@[Fe^III^-TA] (100 μL in PBS, 1.5 × 10^8^ cells) was transfused into the O_2_-purged PBS. The oxygen concentration was recorded over the whole process until the signal was stabilized.

## 4. Conclusions

In summary, we demonstrated that the supramolecular Fe^III^-TA coating on RBC surfaces effectively attenuated the immune response while maintaining the oxygen-carrying capacity. Coordination-driven supramolecular complexation between Fe^III^ and TA was rapid, and made uniform films on individual RBC surfaces, which was confirmed by various characterizations. The coating conditions were optimized for RBCs that are very sensitive to the tonicity of the solution and chemicals. Especially, we found that the use of isotonic saline enhanced the coating efficiency as well as maintained membrane integrity. The chemical motif in supramolecular complex formation of Fe^III^ and TA could be utilized for other materials that contain multivalent catechol groups, such as poly(ethylene glycol) (PEG)-catechol and hyaluronic acid-catechol conjugates [36,37]. Especially, the use of PEG-catechol conjugates for RBC coating would be beneficial in the reduction of potential recognition by immune cells in the body. Considering that the Fe^III^-catechol coating is a rapid, easy, and inexpensive process, we also believe that the formation of RBC-in-shell structures would have a potential in the manufacture of universal RBCs for medical transfusion.

## Supplementary Materials

The following are available online at www.mdpi.com/2073-4360/9/4/140/s1. Figure S1: Optical images of RBCs after 10-min treatment of tannic acid (TA): (a) 0.1 mg mL^−1^, (b) 0.08 mg mL^−1^, and (c) 0.05 mg mL^−1^, Figure S2: Optical images of RBCs after 10-min treatment of FeCl_3_∙6H_2_O: (a) 0.01 mg mL^−1^, (b) 0.005 mg mL^−1^, and (c) 0.001 mg mL^−1^, Figure S3: CLSM image of TA-treated native RBCs after incubation with BSA-Alexa Fluor^®^ 647, Figure S4: Ellipsometric thickness of Fe^III^-TA films on a gold substrate. The films were formed either in water or in isotonic saline (mean ± S.D., *N* = 3).
