# Supplementary material for: Artificial Spores: Immunoprotective Nanocoating of Red Blood Cells with Supramolecular Ferric Ion-Tannic Acid Complex
Source: Polymers (Basel). 2017 Apr 13;9(4):140. doi: 10.3390/polym9040140 (PMC6432373; doi:10.3390/polym9040140)
Supplement: Supplementary file 1 [file polymers-09-00140-s001.pdf]

Supplementary Materials: Artificial Spores: Immunoprotective Nanocoating of Red Blood Cells with Supramolecular Ferric Ion-Tannic Acid Complex

Taegyun Park, Ji Yup Kim, Hyeoncheol Cho, Hee Chul Moon, Beom Jin Kim, Ji Hun Park, Daewha Hong, Joonhong Park and Insung S. Choi

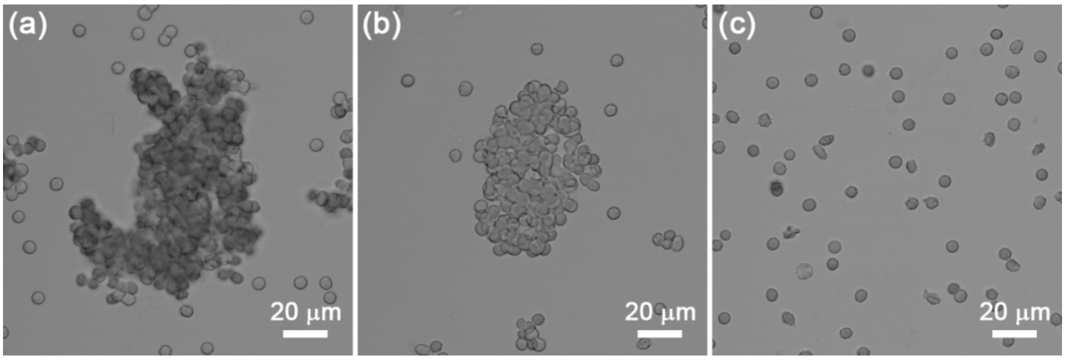

Figure S1. Optical images of RBCs after 10-min treatment of tannic acid (TA): (**a**) 0.1 mg mL^{-1}, (**b**) 0.08 mg mL^{-1}, (**c**) 0.05 mg mL^{-1}.

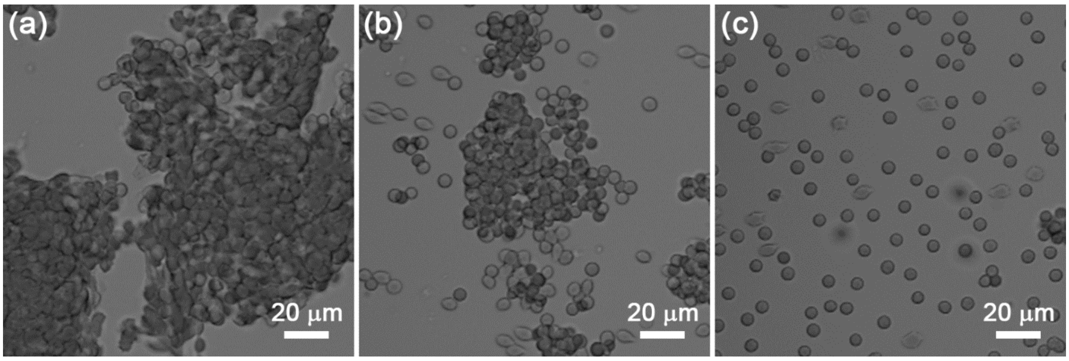

Figure S2. Optical images of RBCs after 10-min treatment of $FeCl_3 \cdot 6H_2O$: (**a**) 0.01 mg mL^{-1}, (**b**) 0.005 mg mL^{-1}, (**c**) 0.001 mg mL^{-1}.

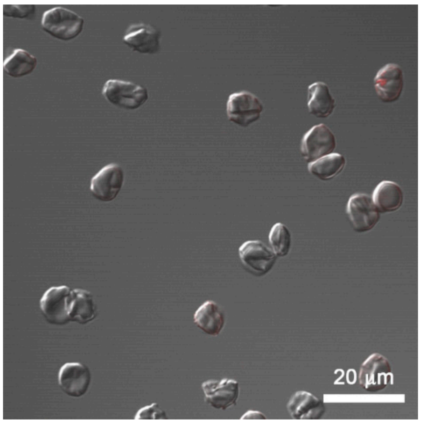

Figure S3. CLSM image of TA-treated native RBCs after incubation with BSA-Alexa Fluor® 647.

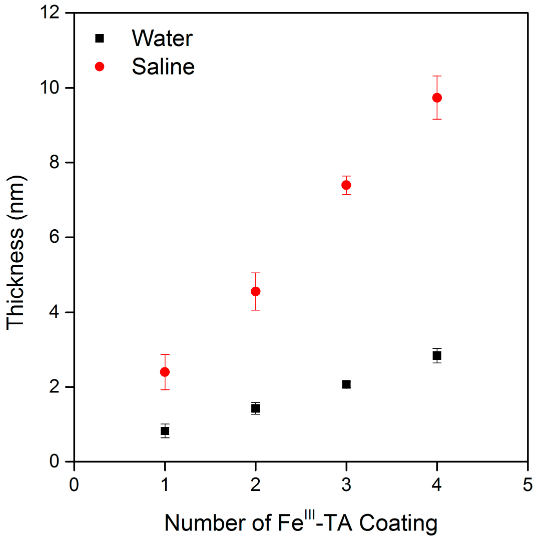

Figure S4. Ellipsometric thickness of Fe^{III}-TA films on a gold substrate. The films were formed either in water or in isotonic saline (mean ± S.D., $N = 3$).
